# Construction of indicator system of tourism professional skills evaluation system in the application-oriented Universities - A case study in Fujian, China

**DOI:** 10.1371/journal.pone.0327785

**Published:** 2025-09-05

**Authors:** Hung-Lung Lin, Xiang Li, Jia-Min Zhang

**Affiliations:** School of Economics and Management, Sanming University, Sanming, Fujian, China; Shandong Jiaotong University, CHINA

## Abstract

This study aims to establish a “Tourism Professional Skills Evaluation System in Application-Oriented Universities” to bridge the gap between industry requirements and traditional curricula. In response to the tourism sector’s rapid growth and shifting market demands, this study employ the Modified Delphi Method (MDM) and Analytic Hierarchy Process (AHP) to identify and weight five core competency domains: basic professional knowledge and skills, professional related knowledge and skills, interpersonal and self-development skills, physical fitness, as well as cultural and ethical rule of ethics qualities. Through expert panels and group discussions, this study develops a comprehensive set of indicators covering all essential competencies. The system has been piloted at a case university and received strong endorsement from both industry and academic stakeholders. By quantitatively defining indicator hierarchies and weights, this framework delivers a scientifically grounded, objective tool for educators, administrators, and policymakers to design and refine tourism talent development programs.

## 1 Introduction

### 1.1 Background and motivation

The implementation of the two-day weekly rest by the Chinese government has led to a significant shift in people’s values, with a growing demand for tourism as a result of the rapid development of China’s market economy and the improvement of people’s living standards. In 2023, domestic tourism revenue soared to 4.91 trillion yuan, marking an extraordinary year-on-year increase of 140.3% [1]. Alternative indicators demonstrate a steady rise in inbound international tourist arrivals over the past three years—from 5.6 million in 2021 to 6.3 million in 2022 (an increase of approximately 12.5%), and further to 7.2 million in 2023 (an increase of roughly 14.3%) [2]. These trends underscore the growing importance of the tourism sector as a vital component of China’s national economy.

The rapid growth of the tourism industry has indeed brought about several problems. One of the most pressing issues is the significant imbalance between the supply and demand of undergraduate tourism professionals. It is concerning to note that, according to data from China’s Ministry of Culture and Tourism [3], there were 35.78 million people engaged in the tourism industry in 2023, but only 65,000 of them held an undergraduate degree in tourism. The significant disparity between supply and demand creates structural contradictions, resulting in employment difficulties for undergraduate tourism graduates. The tourism professional education system is encountering challenges in its development and positioning. The tourism profession has long been viewed as a management or business discipline without an independent theoretical foundation [4–6].

In 2018, China’s Ministry of Education (MOE) established the National Standards for the Quality of Teaching in Tourism Management Professions [7]. The content includes “self-learning ability to acquire and update tourism-related knowledge; basic skills to apply professional knowledge in practice; tourism service and management ability; general skills in information processing operation and application; proficiency in a foreign language should be able to listen, speak, read and write [7]. These major professional development competencies, however, have not made a difference in the development orientation of undergraduate education, such as the training objectives of research-oriented universities are mainly for further education and research while the training objectives of application-oriented universities are mainly for employment. Therefore, the professional training programs set by universities in China are very different from each other, and in general, they seem to be rather arbitrary and lack of strict consistency, which makes it difficult to make vertical and horizontal comparisons. These results will not be conducive to enhancing the employment skill level of undergraduate tourism students, resulting in a more difficult job search expectations for students to achieve. Despite this, undergraduate education has not been significantly impacted, leading to notable disparities and inconsistencies in professional training programs across universities. Thirdly, universities are not adequately preparing students with the skills required by tourism enterprises. Many tourism graduates possess strong theoretical knowledge but lack essential practical skills such as customer service management, digital marketing, and crisis handling—competencies that are highly valued by the industry. For instance, a study by Kim et al. [8] found that employers in the tourism sector often report difficulties in finding graduates who can effectively handle real-world challenges, such as managing guest complaints or utilizing digital tools for tourism promotion. This gap suggests a need for curriculum enhancements that integrate more hands-on training, internships, and industry collaborations to better align with employer expectations. The current higher education professional programs emphasize immediate employment readiness, often prioritizing practical skills for entry-level positions while overlooking the long-term development of middle and senior management competencies. Research by Wakelin-Theron et al. [9] indicates that tourism graduates are well-prepared for operational roles but often lack strategic thinking, leadership, and problem-solving skills essential for managerial positions. While employment orientation is a fundamental goal of professional education, a well-rounded program should balance short-term employability with long-term career growth by incorporating leadership training, critical decision-making exercises, and strategic management coursework. Without this balance, students may struggle to transition into higher-level roles within the industry.

This study will confidently address the following questions: 1. How can we evaluate the core competencies of applied undergraduate tourism students? 2. How can we develop competency standards that meet industry needs? 3. How can we enhance the quality of tourism professional education through an effective assessment system? This study will analyze and discuss the questions above and propose effective solutions to meet the challenges of cultivating tourism talent. Our goal is to promote the sustainable development of university tourism education and the tourism industry.

### 1.2 Innovation and purpose

This study addresses issues in the field of tourism talent development by focusing on the core competencies of applied undergraduate tourism students and formulating industry-relevant competency standards. This innovative study establishes comprehensive assessment criteria to facilitate the design of a targeted curriculum for subsequent university education. The approach aims to ensure that students graduate with the skills needed for the real world, thereby improving the quality of education and students’ competitiveness in employment. The assessment system developed in this study serves as a valuable reference for university educators, administrators, or policymakers in the development of their talent development programs.

### 1.3 Research subjects and scope

This study selects tourism programs within application-oriented universities in Fujian Province, China, as the case for investigation, with the aim of validating the proposed evaluation framework through an in-depth study of a representative region. The rationale for selecting Fujian and the framework’s potential for broader applicability are as follows.

1. Representative tourism resources and formats: Fujian province, located along China’s southeastern coast, boasts rich and diverse tourism assets such as UNESCO heritage sites (e.g., Wuyi Mountain, Taining) and nationally rated 5A attractions like Gulangyu Island. Its tourism offerings span island leisure, ecological vacations, red tourism, and Hakka culture, enabling the study to reflect both common and differentiated competency needs across tourism types.

2. Robust tourism economy: In 2023, Fujian’s tourism revenue surpassed one trillion RMB, with visitor volume and high-star hotel occupancy among the highest in China. This economic scale ensures a solid empirical foundation for analyzing talent demands and validating the evaluation system.

3. Comprehensive higher education structure: Fujian’s higher education ecosystem includes both research- and practice-oriented institutions. Tourism programs are deeply integrated with local market needs, providing an ideal testing ground for aligning academic training with industry expectations.

4. Transferability to other coastal regions: Fujian’s coastal tourism profile is representative of other developed regions such as Jiangsu, Zhejiang, Shanghai, Guangdong, and Hainan. The proposed framework may serve as a reference for talent cultivation and curriculum reform in these areas.

5. Relevance to forest ecotourism areas: Fujian’s experience in wellness-based forest tourism is also applicable to inland regions with similar ecological features, including the Yunnan–Guizhou Plateau, northern Guangxi, and southern Hunan, offering guidance for tourism education in forest-rich areas.

## 2 Literature review

### 2.1 The significance and challenges of the tourism discipline and undergraduate education

The interdisciplinary field of tourism covers a wide range of subjects, such as travel, tourism, culture, geography, sociology, and the environment [10]. As the global tourism industry continues to expand and travelers demand more diverse experiences, universities are increasingly recognizing the educational value of the tourism discipline. Tourism higher education plays a critical role in the development of the tourism industry and the regional economy, as highlighted by its growing significance since the 1990s [11]. Despite the challenges posed by the diversity and specificity of professional tourism disciplines, our expertise and competence in this field allow us to tackle them with confidence and efficiency. Tourism is commonly categorized as a management or business discipline, and as such, many professional programs draw on theoretical knowledge from related fields such as economics, management, and marketing [12,13]. To develop tourism successfully, it is crucial to strike a balance between specialization and diversity in university programs and establish a close linkage with practical industry teaching [14].

Teaching by doing is a crucial extension of the school curriculum that develops students’ practical skills, knowledge, and attitudes to cope with the ever-changing tourism industry [15] Experiencing workplace life outside of school is an essential transition period that minimizes the learning-use gap [16]. Practical teaching improves graduates’ employability by bridging the gap between theory and practice [17]. The effectiveness of practical teaching is influenced by various factors, such as students’ emotions, the surrounding environment, course design, and internship satisfaction [18–20]. A program that combines theory and practice can increase efficiency and shorten training time, thereby reducing costs. Practical experience is crucial for tourism students to successfully find employment and adapt to the work environment. Despite the challenges faced by the tourism industry such as low wages, limited benefits, and high turnover rates, it remains a dynamic and rewarding field [8,21,22].

But, studies have shown that tourism employers tend to choose more repetitive course content when offering off-campus internship programs, which may have a negative impact on students’ learning satisfaction and identification with the industry, which in turn may reduce their willingness to choose a job in the tourism industry [17,20]. Most tourism internship programs tend to be seasonal. For example, during peak tourism seasons, interns are often recruited to fill short-term needs for frontline positions to meet the requirements of the internship program. These short-term jobs cover a wide range of low-level positions such as hotel front office, customer service, food and beverage operations, entertainment, event and conference services, theme parks, attractions, etc. It is important to note that interns typically fill positions that are designed to meet short-term business needs during the local or regional tourist season, rather than based on the need for long-term supervision, management, or leadership training in the organization. This can lead to a decrease in student satisfaction with their learning, which may affect their retention and identification with the profession they are studying, which in turn may reduce their willingness to pursue future employment in the tourism industry [20].

Much of the research on tourism expertise in recent years has focused on how to reform the design of university education programs [13,24–26], and it is important to train middle and lower level managers to meet the expectations of the industry market [8]. To address these issues, Federici et al. [27] suggested that it is important to conduct a survey of the professional skills required by the industry over a period of time to prevent a mismatch between the demand for skills in the labor market and the supply of courses.

### 2.2 Implications and challenges of professional competence, literacy and skills

Birder and Pearson [28] pointed out that the components of professional competence include skills, judgment, attitudes, values, initial skills, knowledge, abilities and aptitudes, etc. Sirdeshmukh et al. [29] considered that professional competence refers to the possession of specialized knowledge and skills in a particular field and the ability to apply them to the workplace, as well as a passion for service and a sense of social responsibility. Professional competence refers to the possession of specialized knowledge and skills in a particular field and the ability to apply them to one’s work, together with a passion for service and a sense of social responsibility. Thus, professional competence refers to the qualification of having sufficient qualitative status or ability to assume a specific role or perform a specific task [30]. “Competence” refers to an individual’s ability to demonstrate consistent behavior, motivation, characteristics, or skills in a particular area, including the knowledge, attitudes, or skills needed to perform a task [31]. In general, competencies can be divided into general competencies, which refer to the knowledge and general skills acquired in everyday life, and specialized competencies, which refer to the specific education or training required to perform a particular job or function [31]. Professional competence includes professional knowledge and skills related to a job or function, and Bowden [32] argues that professional competence includes the knowledge, skills, abilities, and values necessary to perform a professional job, taking into account the potential attributes of the individual, and Perry [33] argues that professional competence must include knowledge, attitudes, and skills that are closely related to job performance and can be improved through training and development. It can also be improved through training and development. According to Jamal et al. [34], six core qualities (technical competence, analytical skills, ecological knowledge, intercultural understanding, ethical and social responsibility, and political awareness) must be integrated into the tourism or hospitality education system to develop core professional skills that meet industry standards. These core skills cover a wide range of elements required in the tourism and hospitality industry, including sustainability, social responsibility, multicultural management, communication skills, innovative thinking, and digital literacy. Nonetheless, according to Kim et al. [8], many companies perceive a gap between the core competencies that hospitality and tourism graduates learn in school and their actual needs, which include caring attitudes, communication skills, integrated thinking skills, language skills, and goal-oriented personalities. This view is consistent with the underlying assumption of this study that there is a mismatch between the skills learned and the skills required by organizations.

### 2.3 Summary of the literature

Firstly, this study explores the importance of tourism as an interdisciplinary field covering a wide range of disciplines for the development of higher education institutions and the tourism industry. However, it also faces the challenge of balancing diversity and professionalism in curriculum design and linkages with industry practice. Second, practical teaching plays an important role in cultivating students’ practical skills and employability, but there are also problems of inadequate curriculum design and repetitive content of internships, which may affect students’ learning satisfaction and industry recognition. Finally, for professional competence, quality and skills, schools must integrate the core professional skills cultivation of industry standards. However, the reality is that there is a gap between the competence that students learn in school and the actual demand, which requires a better match between curriculum content and industry demand.

The main objective of this study is to develop a professional skills assessment system for tourism that can be applied in applied undergraduate universities. The system will be based on the market standards of the travel and tourism industry to provide educators, managers or policy makers with talent development programs and to establish industry-recognized professional skills standards. This will help ensure that tourism professionals are trained to meet the needs of the industry and adapt to the ever-changing market demands and challenges.

## 3 Research methods

### 3.1 Multi-Criteria Decision Making (MCDM) with Delphi Method (DM)

To construct the indicator system, this study first introduces MCDM and its common methods, then draws on Lin et al. [35] to classify decision-support techniques into three categories: 1. objective methods, 2. subjective methods, and 3. hybrid methods—and, on that basis, explains the specific rationale for combining the Modified Delphi Method (MDM) with the Analytic Hierarchy Process (AHP).

MCDM has been widely applied to policy formulation, investment project evaluation, and other complex decision contexts since its introduction, as it can account for multiple evaluation criteria simultaneously [36]. Common MCDM techniques include AHP, TOPSIS, VIKOR, ELECTRE, PROMETHEE, MOORA, entropy, and grey relational analysis, each with distinct data requirements and underlying assumptions [37].

AHP is among the most popular MCDM methods, integrating experts’ qualitative judgments with quantitative weight calculation. It is suitable for long-term forecasting and can conserve research resources when time and funding are limited [36]. However, AHP’s criterion selection is susceptible to subjective bias, potentially destabilizing decision outcomes. To mitigate this limitation, this study introduces the Delphi method (DM)—a systematic expert-consultation technique that uses multiple rounds of anonymous surveys and controlled feedback to reduce individual influence [38]. The MDM enhances the traditional DM by employing structured questionnaires and evaluating indicator importance and consistency via means and quartiles, thereby more efficiently filtering representative elements [39].

Building upon this foundation, Lin et al. [35] categorize decision-support technologies into three types.

1. Objective methods: Objective methods rely on quantifiable and accessible data, such as integer and linear programming, and are applied to tasks like educational budget allocation, timetabling, campus site selection, and digital learning resource optimization [37]. However, education reform also involves nonquantifiable factors—teaching quality, teacher-student interaction, and learning motivation—whose data collection is constrained by time, personnel, and cost, limiting purely objective models’ ability to capture complex educational scenarios.

2. Subjective methods: Represented by MCDM, invite domain experts to provide anonymous ratings and participate in multiple rounds of deliberation, rapidly integrating interdisciplinary insights when problems or evaluation criteria remain unclear [38]. Although subjective methods consume fewer resources than objective analyses, their variables stem from expert judgments and may be influenced by personal preferences; hence, rigorous procedures and consistency checks are required to mitigate bias [39].

3. Hybrid methods: Hybrid methods combine objective and subjective data, using simulation or optimization models to account for both stochastic conditions and expert opinion. When time and funding permit, transforming key decision variables into stochastic parameters and integrating optimization algorithms can yield solutions that are both feasible and robust.

Previous research has successfully integrated DM or MDM with AHP to develop evaluation indicator systems that support policy and program implementation [40–43]. The primary reasons for combining MDM with AHP in this study are threefold.

1. Mitigating subjective bias: MDM’s multi-round, anonymous filtering of elements reduces the subjective bias inherent in AHP criterion selection [36].

2. Adapting to dynamic environments: The tourism industry and educational needs evolve rapidly; purely objective models cannot update promptly, while overly complex simulations exceed available resources. The MDM + AHP approach offers transparency and rapid adjustability.

3. Efficiency and feasibility: AHP effectively structures complex educational management issues into hierarchical levels and assigns quantitative weights to each factor, significantly improving decision-making efficiency and demonstrating strong practical feasibility [40]. MDM’s structured questionnaires and quartile-based filtering further streamline the traditional DM process, substantially reducing time costs [39].

Based on these considerations, this study first employs MDM to establish the indicator system and then utilizes AHP to rank and weight its elements, ultimately constructing a scientifically robust, reliable, and operational indicator system that provides empirical support for educational and managerial decision-making in application-oriented universities.

### 3.2 Research processes

**Fig 1:**
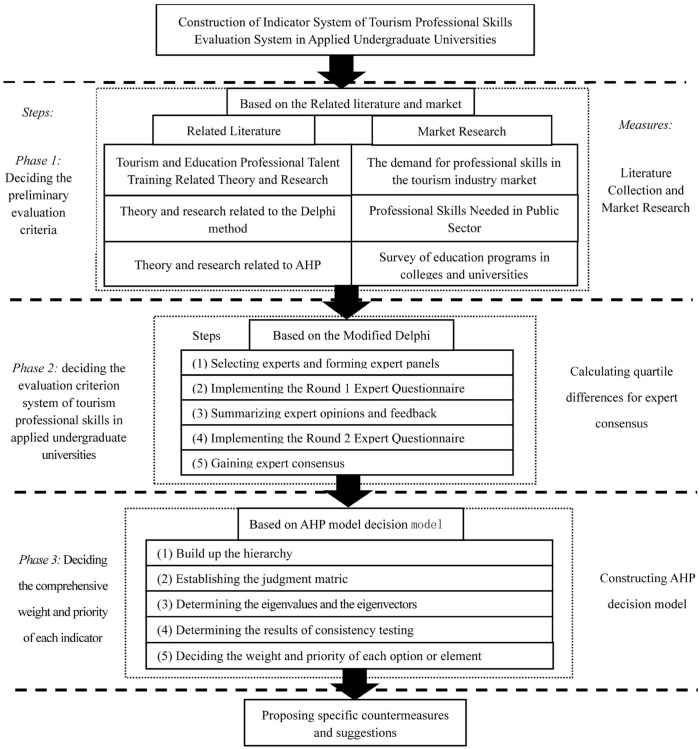
Research framework.

This study is divided into three main phases. The first phase is “deciding the preliminary evaluation criteria”: Based on literature collection and market research, this phase integrates academic research, industry demand, and education supply to collect, compile, and summarize the evaluation criteria and decide the preliminary evaluation criteria. The second phase is “deciding the evaluation criterion system of tourism professional skills in the application-oriented universities”. Based on the guidelines decided in the first phase, this phase conducted the expert opinion solicitation through the MDM, and conduct expert consistency testing using quadratic difference to obtain consensus of the group experts and finally determine the evaluation guideline system. The third phase is “comprehensive weighting and prioritization of each criterion”. In this phase, the evaluation criteria are analyzed by AHP based on the evaluation criteria constructed in the second phase, and specific countermeasures and recommendations are proposed based on the findings of the analysis. The research methodology of this paper is subdivided as follows, and the specific research framework is illustrated in Fig 1.

#### 3.2.1 Phase 1: Deciding the preliminary evaluation criteria: Perspectives based on industry, education and research communities.

1. Consideration of academic views. First, in considering the academic viewpoint, we studied the relevant literature on the cultivation and development of tourism professionals and organize the relevant guidelines that can be used as the basis for evaluation.

2. Consideration of industry market demand and undergraduate talent supply views.

The second step is to find out the positions and skills required for professionals in the travel and tourism industry, taking into account the market demand and the supply view of undergraduate talents.

3. Integrating industry, academia and research to determine preliminary evaluation guidelines.

In the third step, the first two steps were synthesized and summarized to determine the initial evaluation criteria.

#### 3.2.2 Phase 2: Deciding on initial evaluation guidelines - based on a Modified Delphi Method (MDM).

According to Murry and Hammons [44], the DM is an anonymous expert group decision making technique that uses the experience and expertise of experts in a particular issue, and through multiple rounds of iterative consultation and feedback, to achieve a consensus on the issue.

Based on the analysis of DM by Rowe and Wright [45] and MDM by Lin and Ma [39], the primary processes of these methods are summarized as follows: (1) Anonymity – The survey is conducted in the form of a questionnaire, which enhances the independence of individual experts in providing responses. Since opinions are expressed privately rather than in a group discussion, the influence of authority figures and social pressure—whether in the form of dominant opinions, arbitrary personal views, or majority influence—is significantly reduced. (2) Repeatability–Multiple rounds of questionnaire responses allow experts to refine their opinions over time. Experts can adjust their judgments without fear of social pressure, ensuring a more thoughtful and independent decision-making process. (3) Controlled Feedback – After each round of questionnaire responses, the researcher (mediator) compiles and summarizes expert opinions before redistributing them for further refinement. Unlike direct discussions, feedback is structured, allowing experts to consider collective insights in a controlled manner. (4) Consensus Building–The process continues until expert opinions converge on a consensus. Since DM primarily relies on open-ended questionnaires, the researcher (mediator) must systematically collect, organize, and synthesize expert feedback before redistributing it. This iterative process is labor-intensive and time-consuming.

The Modified Delphi Method (MDM) builds upon the fundamental structure of DM but introduces key modifications. While the overall process remains similar, MDM replaces the open-ended questionnaire with a structured questionnaire while still allowing room for open-ended comments. In Step 3 of the process, statistical summaries—such as mean values, mode, standard deviation, or quartile deviation (QD)—are incorporated to present expert opinions more systematically.

Furthermore, according to Lin and Ma [39], the implementation of MDM requires expert recommendations from representatives who are well-versed in the field, including professionals from industry, academia, and government sectors. Each expert must have at least five years of experience in the relevant domain. A minimum of nine experts is required to ensure a diverse and well-informed panel. Additionally, one researcher (mediator) is responsible for distributing the questionnaires, compiling expert opinions, and providing feedback to the panel. Therefore, the MDM process requires at least nine experts and one researcher (mediator), totaling ten participants.

#### 3.2.3 Phase 3: Deciding the comprehensive weight and priority of each indicator - based on Analytic Hierarchy Process (AHP) decision model.

The AHP proposed by Saaty in 1980, is a widely utilized decision-making method designed to address complex problems within social systems. It has been extensively applied across various disciplines, including engineering, economics, and management [46]. AHP is based on the fundamental assumption that evaluation criteria are mutually independent. However, in real-world scenarios, these criteria often exhibit interdependencies and reciprocal influences [47].

To address this limitation, Saaty introduced the Analytic Network Process (ANP) in the 1996, which accounts for complex interrelationships among factors within a network structure. While ANP offers a more realistic representation of decision-making environments and has been shown to enhance the accuracy of evaluation outcomes compared to AHP [39] its implementation poses several challenges: (1) Computational complexity: ANP requires the construction of multiple judgment matrices and the computation of a supermatrix, which significantly increases computational complexity, particularly as the number of decision factors grows. (2) Consistency verification: AHP includes a well-defined consistency check mechanism to ensure logical coherence in pairwise comparisons, thereby minimizing subjective bias. In contrast, ANP’s reliance on supermatrix calculations complicates consistency verification, potentially increasing the impact of subjective influences. (3) Applicability and interpretability: AHP’s hierarchical structure and weight computation methodology provide clear interpretability, facilitating the visualization and communication of results. Conversely, ANP’s network-based structure and intricate calculations may reduce its practical applicability and acceptance among decision-makers.

To further examine the implications of these methodological differences, Kasirian et al. [48] conducted a comparative study on supplier selection in the automotive manufacturing sector, applying both AHP (assuming independence) and ANP (considering interdependencies). Additionally, they employed Preemptive Goal Programming with two objectives: maximizing the Total Value of Purchasing and minimizing the Total Cost of Purchasing (TCP), to determine the optimal approach. Their findings indicated that while ANP yielded more precise results, the difference in TCP values between the two methods was negligible.

Considering these factors, this study adopts AHP as the primary methodological framework due to its computational efficiency, practical applicability, and well-established consistency verification mechanism. First, the decision maker establishes the overall goal for his or her problem, and then develops sub-goals (i.e., lower-level elements) from the characteristics of the overall goal until the final layer of elements is constructed and the hierarchy is mapped out. After that, based on the constructed hierarchy diagram, a two-by-two judgment matrix is established for each level and evaluated on a scale from 1 to 9. The eigenvectors of each judgment matrix are then derived as the weights of each evaluation element. Eventually, the priority order of the solutions or elements is obtained after comprehensive weighting. The establishment of the hierarchy is a significant part of the AHP application, which simplifies complex problems and enables decision makers to make the right decisions more conveniently. Its calculation steps are described as follows [46].

Step 1: Creating a hierarchy.

When building a hierarchy, there is no certain standard procedure for constructing the hierarchy. The highest level of the hierarchy is the ultimate goal of the evaluation problem, and the lowest level is usually the solution.

Step 2: Establishing the judgment matrix.

The hierarchical analysis method mainly involves using the factors on the upper level of each level as the basis for comparing the evaluation of factors on this level, and then making a judgmental comparison of factors. If there are *n* factors within the hierarchy, n(n−1)/2 judgments are required for comparison. This approach is developed to simplify the complexity of the problem so that decision makers can focus on the relationship between the two factors. The assessment items are two-by-two comparisons with other assessment factors within the same tier on a 9 scale under the assessment benchmark of the assessment items in the previous tier. The judgment matrix *A* is established, and $C_{1},C_{2},\ldots ,C_{n}$ is a set of elements, and the judgment of the quantification of element $C_{i},C_{j}$ can be expressed as a *n*-by-*n* matrix *A* as follows:

$$A=(a_{ij})=(\begin{array}{cccc} 1 & a_{12} & L & a_{1n}\\ 1/a_{12} & 1 & L & a_{2n}\\ \ldots & \ldots & \ldots & \ldots \\ 1/a_{1n} & 1/a_{2n} & L & 1 \end{array})$$ (1)

When $a_{ij}=\frac{1}{a_{ij}}=i,j=1,2,3\ldots ,n$, given to pairs of two elements $\left(C_{i},C_{j}\right)$ a quantified relative importance judgment in the matrix *A*, presented by the value *a*_*ij*_, expressed by $W_{1},W_{2},\ldots ,W_{n}$ as the weight of the quantification of *n* elements $C_{1},C_{2},\ldots ,C_{n}$, can react to the recorded judgment values.

Step 3: Determining the eigenvalues and the eigenvectors. The judgment matrix *A* multiplied by the weight vector *x* of the elements is equal to *nx*, that is, (A−nI)x=0. *x* is then the eigenvector of the eigenvalue *n*. Since *a*_*ij*_ is a subjective judgment given by the decision maker when making pairwise comparisons, there must be some degree of difference with the true value of $W_{i}/W_{j}$, so *Ax* = *nx* is not valid, and Saaty [44] suggests replacing *n* with the maximum eigenvalue $\lambda_{max}$ of the *A* matrix, which is illustrated in the following part.

$$\lambda_{max}=\sum_{j=1}^{n}a_{ij}\frac{W_{j}}{W_{i}}$$ (2)

In case of Consistency matri, the feature vector *x* can be calculated by (3).

(A−nI)x=0 (3)

Step 4: Determining the results of consistency testing. AHP uses Consistency Index (*C*.*I*.) to measure the consistency of paired judgment matrices in order to correct for unreasonable assessment values. *C*.*I*. is defined as in Equation (4).

$$C.I.=\frac{\lambda_{max}-n}{n-1}$$ (4)

C.I. = 0 indicates that there is complete consistency between the previous and subsequent judgments, and Saaty [45] suggests that C.I.≤0.1 is an allowable bias. Table 1 shows that different values of n produce different *C*.*I*. values, which is called Random Index (*R*.*I*.), and the ratio of *C*.*I*. values to *R*.*I*. values for the same matrix of n values is called Consistency Ratio (*C*.*R*.).

$$C.R.=\frac{C.I.}{R.I.}$$ (5)

If C.R.≤0.1, then consistency is satisfied.

Step 5: Deciding the weight and priority of each option or element. After calculating each weight among the elements of each level, the overall level weights are then calculated. In the end, the weight and priority of each option or element is decided, which is an important reference for decision makers to make decisions or plans.

**Table 1:** Table of random indicator values of *n* order positive inverse matrix.

*n*	1	2	3	4	5	6	7	8	9	10	11
*R*.*I*.	0.00	0.00	0.58	0.90	1.12	1.24	1.32	1.41	1.45	1.49	1.51

### 3.3 Ethical considerations

This study did not involve medical or clinical research requiring institutional review board approval. All expert participants provided informed consent and signed consent forms prior to completing the questionnaire survey.

## 4 Results and findings

The main purpose of this study is to construct “Tourism Professional Skills Evaluation System in The Application-oriented Universities”, and the research analysis was implemented based on three phases. The specific implementation steps of each phase are described below.

### 4.1 Phase 1: Deciding the preliminary evaluation criteria: Perspectives based on industry, education and research communities

This study integrates insights from academic research, industry demand, and education supply perspectives to establish preliminary evaluation criteria. The process involved literature collection and market research from November 2020 to February 2021, focusing on the training and development of tourism professionals, required skills for travel and tourism industry positions, and training programs in undergraduate education. Based on these sources, a comprehensive set of evaluation criteria was formulated, leading to the development of a modified Delphi questionnaire for further refinement. This phase consists of three steps, detailed as follows:

Step 1: Academic Research-Based Indicator Selection.

To incorporate academic perspectives, this study conducted a systematic literature review using domestic and international academic databases. From November to December 2020, 35 relevant research papers on tourism management education, professional development, and competency frameworks were reviewed. The findings from these studies provided a theoretical foundation for indicator selection, particularly in defining the key competencies required for tourism graduates. This step resulted in the initial identification of evaluation criteria grounded in established academic frameworks.

Step 2: Industry Market Demand and Undergraduate Talent Supply.

To ensure alignment with industry needs and educational training, this study analyzed job recruitment websites and undergraduate tourism-related programs in China and Taiwan from November to December 2020. (1) Job Market Analysis: The study examined postings for middle and senior management positions in the travel and tourism industry, including tour guides, marketing, finance, and sales managers in travel agencies, as well as front desk managers, marketing and sales, finance, human resource management, and room and food service managers in hotels. This review identified the core skills and qualifications expected by employers in these fields. (2) Undergraduate Program Review: A detailed review was conducted on tourism, leisure, hospitality, catering, and shipping management programs at universities in China and Taiwan. The analysis focused on curriculum structures, learning objectives, and course components to determine the alignment between academic training and industry expectations.

By integrating job market demand analysis and undergraduate curriculum review, a total of 62 subcriteria were identified, forming the basis for evaluating tourism professionals’ competencies. These subcriteria were classified into seven major competency categories that encompass both industry and educational perspectives, ensuring a comprehensive evaluation framework.

Step 3: Integration of Industry, Academia, and Research.

Based on the 62 subcriteria identified in Steps 1 and 2 and classified under seven major competency categories, two expert meetings were conducted from January to February 2021. These meetings gathered five university professors specializing in tourism education, each with over 10 years of teaching experience, and four senior industry professionals from the tourism and hotel sectors, each with over 10 years of management experience. The discussions aimed to refine and consolidate the evaluation indicators, ensuring they comprehensively reflected both academic and industry perspectives. Through repeated discussions and revisions, the evaluation framework was finalized into five major criteria and 47 subcriteria, categorized as follows: *C*_1_: Fundamental professional knowledge and skills – 11 subcriteria, *C*_2_: Professional-related knowledge and skills – 10 subcriteria, *C*_3_: Interpersonal relationship and self-development ability – 10 subcriteria, *C*_4_: Cultural, ethical, and legal literacy – 8 subcriteria and *C*_5_: Physical fitness – 8 subcriteria.

These refined indicators formed the basis for the preliminary MDM questionnaire, which was used for further validation and expert consensus-building.

### 4.2 Phase 2: Deciding on initial evaluation guidelines - based on MDM

**Fig 2:**
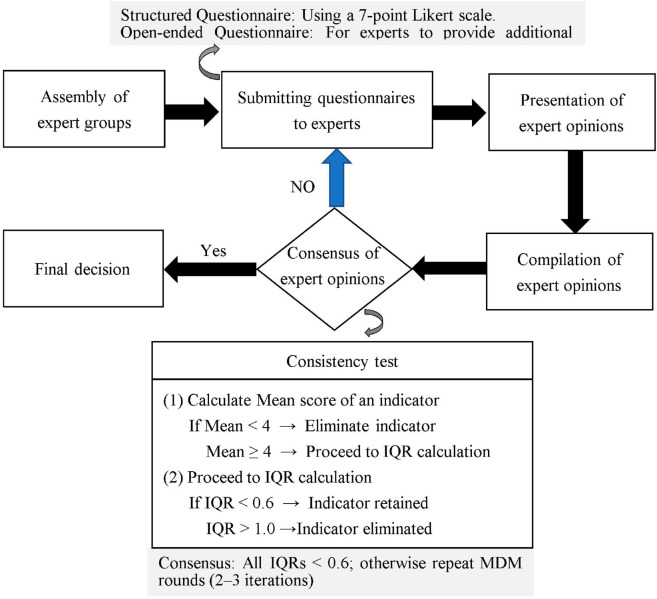
Process for MDM implementation.

This study rigorously followed the MDM procedures outlined by Lin and Ma (2021) to conduct the survey. The complete data processing workflow is illustrated in Fig 2. The specific execution steps are as follows.

Step 1: Design the questionnaire.

Before implementing the Modified Delphi Method (MDM), an initial screening and sorting of evaluation guidelines were conducted. This process led to the selection of 47 initial guidelines. These guidelines were derived from three key sources: (1) literature review, (2) curriculum analysis of undergraduate tourism-related programs in China and Taiwan, and (3) industry job market analysis. The selected guidelines were then used as the foundation for designing the questionnaire. Based on this, the first-round questionnaire was developed.

Step 2: Form an expert team.

A structured Likert-scale questionnaire (ranging from 1 = “very unimportant” to 7 = “very important”) was developed based on the selected indicators. To ensure methodological rigor and validity, a total of 18 experts from both industry and academia were carefully selected to participate in the MDM survey. These experts were chosen for their extensive professional experience in the tourism and hospitality sectors, thereby ensuring a balanced integration of academic insight and practical perspective.

In order to further guarantee both regional and sectoral representativeness, experts were drawn from government agencies, higher education institutions, and enterprises (including both travel and hotel sectors) located in five major tourism cities in Fujian Province: Fuzhou, Xiamen, Quanzhou, Zhangzhou, and Sanming. The composition of the expert panel is as follows.

1. Tourism Industry Experts (9 participants). (1) Senior executives (5 experts): These individuals hold director-level or higher positions in leading travel agencies and online travel service platforms across the five aforementioned cities. Each possesses more than five years of experience in corporate operations and strategic management. (2) Professional tour guides (4 experts): These experts are certified national first- or second-class tour guides based in Fuzhou, Xiamen, and Quanzhou. With over three years of frontline experience, they are highly proficient in group reception, itinerary design, and tourism product development.

2. Hospitality industry experts (4 participants). These experts are currently serving as department managers or deputy operations directors in reputable chain hotels located in Xiamen, Fuzhou, and Sanming. All have more than five years of experience in managing four-star hotel operations, providing valuable insight into service quality and hotel management competencies.

3. Academic experts (5 participants). These faculty members are affiliated with tourism management programs at universities in Fuzhou, Xiamen, and Sanming. All hold the rank of associate professor or above, with at least ten years of experience in teaching, research, and academic-industry collaboration in the field of tourism.

This multi-dimensional expert panel ensured that the evaluation indicators were reviewed from diverse professional standpoints and within a representative regional context, thereby enhancing the credibility and applicability of the study’s findings.

Step 3: Detection of expert consistency.

Following the theoretical foundations of Lin and Ma [39] and Hollden and Wedman [49] on Delphi Method (DM) and Modified Delphi Method (MDM), this study examined expert agreement using the interquartile range (IQR). The consensus criteria were as follows: (1) If the mean score of an indicator was below 4, it was deemed not sufficiently important and eliminated. (2) If the IQR was below 0.6, the indicator was retained, as it demonstrated a high degree of expert agreement. (3) If the IQR exceeded 1.0, the indicator was eliminated, as it indicated a lack of consensus among experts.

To ensure comprehensive feedback and expert participation, the MDM questionnaire was distributed via in-person delivery, email, and WeChat. After each round, the responses were analyzed and refined before proceeding to the next round. The entire Delphi survey process was conducted from March to April 2021, with two rounds of revisions.

After two rounds of MDM questionnaire surveys, the study ultimately established the “Tourism Professional Skills Evaluation System for Application-Oriented Universities”, which was structured into five major criteria and 28 sub-criteria (see Appendix Table A). These five major criteria were derived through a combination of expert input, literature review, and categorization of the 28 sub-criteria, ensuring that the final framework aligned with both industry demands and academic standards.

### 4.3 Phase 3: Deciding the combined weight and priority of each criterion - based on AHP multi-criteria decision model

A systematic analysis was performed in this phase based on the criteria decided by the second phase of the MDM with the AHP decision model, and the comprehensive weight and priority of each criterion was decided by AHP, and the detailed analysis steps are described below.

Step 1: Create a hierarchy.

**Fig 3:**
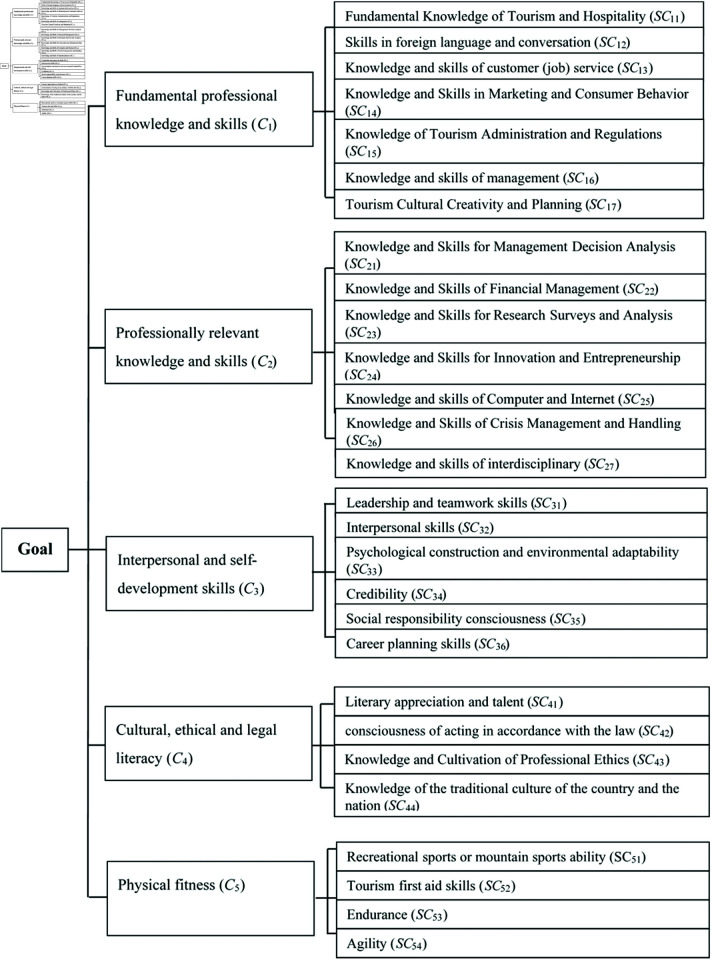
Hierarchical structure of the tourism professional skills evaluation system in the application-oriented universities.

The hierarchy was constructed according to the basic assumptions of AHP based on the detection results of the MDM quartiles in Appendix: Table A, which can be divided into 5 levels (criteria) and 28 sub-criteria, and the definition of sub-criteria for professional skills are in Appendix: Table B. The hierarchical structure of tourism professional skills evaluation system in the application-oriented universities is shown in Fig 3.

Step 2: Establish the judgment matrix.

A total of 18 AHP expert questionnaires were distributed according to the hierarchy constructed in Step 1, and the composition of the experts consisted of representatives from industry, government and academia. There are five representatives from the industry who are department directors or general managers in the industry and are proficient in human resource management of high-starred hotels and management of tourist attractions as well as travel agencies. There are 8 experts from government units who are engaged in natural heritage, tourism and cultural administration in government departments and are proficient in world natural heritage conservation, tourism site brand promoting, cultural tourism festival event planning, and tourism project investment attraction. There are five representatives from academia, and they work as teachers in tourism-related fields in universities, and are proficient in tourism geography, sustainable tourism management, hotel management, rural tourism and leisure agriculture. The questionnaire was implemented from May 03, 2021 to May 10, 2019. The questionnaire design in this study adopts the 9-point scale proposed by Saaty [46]. First, experts conducted pairwise comparisons of the criteria at the primary level. Next, within each primary criterion, they performed pairwise comparisons of the secondary criteria. Based on these evaluations, judgment matrices were sequentially constructed for each hierarchical level as the foundation for subsequent analyses. Following Saaty’s [46] recommendation, the geometric mean method was used to aggregate individual expert judgments. Accordingly, after verifying that each expert’s judgment matrix satisfied the consistency requirements, we applied the geometric mean to integrate all expert matrices and then constructed the final aggregate judgment matrix according to equation (1).

Step 3: Decide on the eigenvalues and eigenvectors.

This step is applied to calculate each judgment matrix constructed in Step 2 and find the eigenvectors (weights) of each matrix based on equations (2) to (3). Table 2 presents the relative weights of the main criteria (dimensions), and Tables 3 to 7 present the relative weights of each sub-criteria.

**Table 2:** Judgment matrix and relative weights of the main criteria of the evaluation system.

	$C_{1}$	$C_{2}$	$C_{3}$	$C_{4}$	$C_{5}$	Eigenvectors
*C*_1_	1.000	1.346	2.059	3.889	2.692	0.338
*C*_2_	0.743	1.000	3.231	1.636	2.312	0.286
*C*_3_	0.486	0.310	1.000	2.554	1.308	0.157
*C*_4_	0.257	0.611	0.392	1.000	0.693	0.097
*C*_5_	0.371	0.433	0.765	1.444	1.000	0.122

$\lambda_{max}$ = 5.192, C.I. = 0.05, R.I. = 1.12, C.R. = 0.04.

**Table 3:** udgment matrix and relative weights under fundamental professional knowledge and skills.

	$SC_{11}$	$SC_{12}$	$SC_{13}$	$SC_{14}$	$SC_{15}$	$SC_{16}$	$SC_{17}$)	Eigenvectors
*SC*_11_	1.000	4.333	1.346	2.889	5.210	1.444	2.167	0.252
*SC*_12_	0.231	1.000	0.403	0.408	2.361	0.317	0.321	0.062
*SC*_13_	0.743	2.480	1.000	2.432	2.571	2.143	3.415	0.234
*SC*_14_	0.346	2.451	0.411	1.000	2.154	0.432	0.407	0.088
*SC*_15_	0.192	0.424	0.389	0.464	1.000	0.242	0.348	0.047
*SC*_16_	0.693	3.154	0.467	2.315	4.125	1.000	3.145	0.195
*SC*_17_	0.461	3.112	0.293	2.457	2.874	0.317	1.000	0.122

$\lambda_{max}$ = 7.445, C.I. = 0.074, R.I. = 1.32, C.R. = 0.056.

**Table 4:** Judgment matrix and relative weights under profession-related knowledge and skills.

	$SC_{21}$	$SC_{22}$	$SC_{23}$	$SC_{24}$	$SC_{25}$	$SC_{26}$	$SC_{27}$)	Eigenvectors
*SC*_21_	1.000	3.286	2.242	1.878	2.925	1.782	4.582	0.277
*SC*_22_	0.304	1.000	0.429	0.389	0.600	0.350	2.015	0.070
*SC*_23_	0.446	2.333	1.000	0.778	2.114	0.700	2.333	0.141
*SC*_24_	0.532	2.571	1.286	1.000	2.415	0.552	3.600	0.165
*SC*_25_	0.342	1.667	0.473	0.414	1.000	0.415	3.154	0.094
*SC*_26_	0.561	2.857	1.429	1.811	2.412	1.000	4.120	0.207
*SC*_27_	0.218	0.496	0.429	0.278	0.317	0.243	1.000	0.046

$\lambda_{max}$ = 7.147, C.I. = 0.024, R.I. = 1.32, C.R. = 0.019.

**Table 5:** Judgment matrix and relative weights under the competencies of interpersonal relationships and self-development.

	$SC_{31}$	$SC_{32}$	$SC_{33}$	$SC_{34}$	$SC_{35}$	$SC_{36}$	Eigenvectors
*SC*_31_	1.000	3.415	2.881	0.788	4.125	3.884	0.296
*SC*_32_	0.293	1.000	0.355	0.303	2.771	2.512	0.105
*SC*_33_	0.347	2.815	1.000	0.485	3.154	2.642	0.168
*SC*_34_	1.269	3.300	2.063	1.000	6.600	3.667	0.310
*SC*_35_	0.242	0.361	0.317	0.152	1.000	0.321	0.045
*SC*_36_	0.257	0.398	0.379	0.273	3.115	1.000	0.076

$\lambda_{max}$ = 6.292, C.I. = 0.058, R.I. = 1.12, C.R. = 0.052.

**Table 6:** Judgment matrix and relative weights under Cultural, ethical and legal literacy.

	$SC_{41}$	$SC_{42}$	$SC_{43}$	$SC_{44}$	Eigenvectors
*SC*_41_	1.000	0.320	0.410	1.889	0.145
*SC*_42_	3.122	1.000	0.539	3.882	0.332
*SC*_43_	2.438	1.854	1.000	4.444	0.438
*SC*_44_	0.529	0.258	0.225	1.000	0.085

$\lambda_{max}$ = 4.037, C.I. = 0.024, R.I. = 0.9, C.R. = 0.027.

**Table 7:** Judgment matrix and relative weights under physical fitness.

	$SC_{51}$	$SC_{52}$	$SC_{53}$	$SC_{54}$	Eigenvectors
*SC*_51_	1.000	0.275	0.417	0.396	0.100
*SC*_52_	3.636	1.000	1.538	2.667	0.416
*SC*_53_	2.400	0.650	1.000	2.846	0.317
*SC*_54_	2.524	0.375	0.351	1.000	0.167

$\lambda_{max}$ = 4.116, C.I. = 0.039, R.I. = 0.9, C.R. = 0.043.

Step 4: Decide on the results of consistency testing.

To ensure consistent results from the experts in this study, the questionnaire was administered to each of the 18 experts in a one-on-one above-the-line face-to-face manner to ensure that the judgement matrices constructed by each expert were consistent with the results of the test. If there is a discrepancy in the results, the experts were asked to complete the questionnaire again until the results are correct. This step determines the consistency test results of each judgment matrix based on equations (4) and (5), and the *C*.*I*. and *C*.*R*. values obtained from each matrix are < 0.1. Therefore, the results of the judgment matrices constructed in this paper are consistent with the principle of consistency test, and the *C*.*I*. and *C*.*R*. are displayed in Tables 2 to 7.

Step 5: Decide the weighting and priority of each option or element.

The integrated weights of the overall criteria were calculated by weighting the weights of the main criteria with the weights of the sub-criteria, and the results are shown in Table 8.

**Table 8:** Results of the evaluation of the indicator system.

Criteria	Weighting	Sub-criteria	Weighting	Overall Integrated Weighting	ranking
*C*_1_	0.338	*SC*_11_	0.252	0.085	1
		*SC*_12_	0.062	0.021	18
		*SC*_13_	0.234	0.079	2
		*SC*_14_	0.088	0.030	15
		*SC*_15_	0.047	0.016	21
		*SC*_16_	0.195	0.066	4
		*SC*_17_	0.122	0.041	11
*C*_2_	0.286	*SC*_21_	0.277	0.079	2
		*SC*_22_	0.07	0.020	19
		*SC*_23_	0.141	0.040	12
		*SC*_24_	0.165	0.047	8
		*SC*_25_	0.094	0.027	16
		*SC*_26_	0.207	0.059	5
		*SC*_27_	0.0462	0.013	24
*C*_3_	0.157	*SC*_31_	0.296	0.046	9
		*SC*_32_	0.105	0.016	21
		*SC*_33_	0.168	0.026	17
		*SC*_34_	0.31	0.049	7
		*SC*_35_	0.045	0.008	28
		*SC*_36_	0.076	0.012	25
*C*_4_	0.097	*SC*_41_	0.145	0.014	23
		*SC*_42_	0.332	0.032	14
		*SC*_43_	0.438	0.042	10
		*SC*_44_	0.085	0.010	27
*C*_5_	0.122	*SC*_51_	0.100	0.012	25
		*SC*_52_	0.416	0.051	6
		*SC*_53_	0.317	0.039	13
		*SC*_54_	0.167	0.020	19

### 4.4 Results and findings

According to Table 8, the sum of the weight of the two criteria of fundamental professional knowledge and skills *C*_1_ and professional-related knowledge and skills *C*_2_ is 0.624, which is slightly higher than 60%. This indicates that students of tourism majors in the application-oriented universities should master solid professional foundation or professional-related knowledge and skills through learning and participation in practical training and internship, so as to lay a solid base for quick adaptation to work and quicker performance after graduation, and then get promotion opportunities. The interpersonal relationship and self-development ability *C*_3_ ranked in the middle of the five criteria. In the process of tourism enterprises to carry out business management and customer service, they need to properly manage the relationship between employees, between employees and supervisors, between the professional manager team and the owner side, and between employees and customers. Only then can the tourism business function properly. For this reason, the students of tourism majors should not only focus on mastering the basic and professional-related knowledge and skills, but also pay moderate attention to the learning of interpersonal relationship and self-development level knowledge and skills, and reserve the relevant knowledge during their study. Physical fitness *C*_5_ has a slightly higher criteria weighting than Cultural, ethical and legal literacy *C*_4_. From the feedback of undergraduate tourism students’ internship in the application-oriented universities, the work intensity of internship in direct-to-customer service departments such as rooms and catering in five-star hotels would be greater. The working hours are generally extended especially during the peak season, and the internship students generally report to their internship supervisors that their work is very stressful. Thus, working in the tourism business requires a good physical fitness level. Cultural, ethical and legal literacy *C*_4_ ranked at the end of the five guidelines, which indicates that the current stage of development of Chinese tourism enterprises, culture, morality and rule of law aspects have not drawn sufficient attention. Maintaining a passing level in this respect is fine enough. However, it is predictable that this criterion would become increasingly important for companies in the tourism industry to maintain sustainable development and to promote high-quality development on this basis. In this way, the students should also spend the necessary time and effort to learn about it.

The overall comprehensive weighting of the sub-criteria is ranked from highest to lowest, and the top 10 was highlighted in Table 8. Fundamental knowledge of tourism and hospitality *SC*_11_ is a compulsory content for undergraduate tourism majors, and it is the essential knowledge that students should have to work in tourism industry after graduation, which plays a decisive role. Customer (job) service knowledge and skills *SC*_13_ is for particular jobs and is a concrete application of basic knowledge of the tourism industry. From the perspective of career competency, the students of tourism should try to master the knowledge and skills of the proposed job after graduation, so as to shorten the time to adapt to the workplace. Knowledge and skills for management decision analysis *SC*_21_ and knowledge and skills for customer (job) service *SC*_13_ have the same overall integrated weighting. The tourism majors in the application-oriented university focus on training high-quality management talents in tourism industry, and decision analysis is the starting point of all management work. It is crucial to make correct decisions, especially under the complex and changing conditions of the external environment. The Knowledge and Skills of Management *SC*_16_ focuses on the ability to execute, which is the key link between the decision making intention and whether the decision target can be achieved in quality and quantity on time, and is also an important element for training professional managers in the tourism industry. The negative impact of the global outbreak of New Crown Pneumonia in 2020 on the tourism industry has been particularly severe, with some tourism enterprises in shutdown or bankruptcy difficulties. In addition, tourism enterprises are too competitive and many small and micro tourism enterprises may fail if there are unfavorable external environmental factors. Hence, students should firmly develop and acquire the knowledge and skills of crisis management and handling during their studies *SC*_26_. Ensuring the safety of tourists is the lifeline of the tourism industry. Due to sudden natural disasters (e.g. earthquakes, sudden heavy rainfall, landslides, etc.), human factors (fires, traffic accidents due to carelessness) and tourists’ own reasons (such as sudden illness), it is necessary for tourism staff to master first aid skills *SC*_52_. Tourism students should acquire progressive skills including but not limited to CPR, simple medical bandaging, survival skills (earthquake, fire, flash flood, mudslide, and so on). Credibility *SC*_34_ is an important cornerstone to maintain a good image and market reputation of tourism enterprises. It is particularly important for travel enterprises to properly handle the refund of advance payment when the contract cannot be fulfilled due to force majeure or unexpected factors. The employees of tourism companies are expected to keep their promises when they are students. From the perspective of matching supply and demand, as the demand of tourists presents a niche and variable character, there is no solution to better meet the demand of tourists except to provide new supply through innovation and entrepreneurship. Consequently, tourism students should have sufficient and useful knowledge and skills in innovation and entrepreneurship *SC*_24_. Decision-making, business management or medium to large service activities to achieve the desired goal requires both an excellent leader and guide and the power of teamwork. Accordingly, undergraduate tourism students should be competent in leadership and teamwork *SC*_31_. Knowledge and cultivation of professional ethics *SC*_43_ is an eternal necessity for tourism professionals, especially professional managers of tourism enterprises. The faculties should repeatedly emphasize the importance of adhering to professional ethics.

## 5 Discussion and conclusion

### 5.1 Discussion

Based on the previous literature review and the results of the current study, we can conduct a more in-depth exploration of the development direction and training needs of professional tourism education, focusing mainly on its theoretical and practical aspects, i.e. its theoretical and practical significance. The importance of these two aspects will be discussed separately below.

#### 5.1.1 Foundation.

1. Methodological Support for the Construction of the Indicator System.

This study’s evaluation method is based on the MCDM, primarily utilizing MDM and AHP to construct the indicator system and determine its weightings. (1) Theoretical support for MDM: MDM originates from decision science and expert consensus theory. Its core principle involves collecting and refining expert opinions anonymously over multiple rounds to minimize individual expert influence and enhance decision-making objectivity and accuracy [39]. In this study, MDM is applied to ensure the authority and rationality of the core competency indicators in tourism education while mitigating biases from individual experts. (2) Theoretical Support for AHP: AHP is a decision-making analysis method proposed by Saaty [46]. Based on structured psychological and mathematical models, it systematically ranks indicators through hierarchical classification and pairwise comparisons, quantifying the relative importance of different factors. This study employs AHP to determine the weight of each core competency indicator, ensuring that the evaluation system is quantifiable and comparable, thereby enhancing the reliability and practical applicability of the results.

2. Theoretical Support for the Construction of the Indicator System.

The indicator system in this study is developed based on various educational and talent development theories to ensure that the established core competencies align with the training objectives of application-oriented undergraduate tourism programs.

(1) Core competencies theory (CCT): Hamel & Prahalad [50] proposed theCCT, emphasizing that organizations or individuals should possess key capabilities that provide long-term competitive advantages. In the context of application-oriented undergraduate education, this study posits that students should acquire core competencies in fundamental professional knowledge and skills (*C*_1_), professional-related knowledge and skills *C*_2_, interpersonal and self-development skills *C*_3_, cultural, ethical, and legal literacy *C*_4_, and physical fitness *C*_5_ to meet industry development demands. According to Makulova et al. [51], competency-based education emphasizes that students should develop diverse abilities to navigate complex work environments. This perspective informs the comprehensive approach of this study’s indicator system, which highlights both technical skills and soft skills. Specifically, the designed indicators cover technical knowledge and skills (*C*_1_ and *C*_2_) as well as soft skills (*C*_3_: interpersonal and self-development, *C*_4_: cultural and legal literacy, and *C*_5_: physical fitness) to fully meet the unique requirements of the tourism industry.

(2) Competency-based education (CBE): CBE focuses on measurable and operational competency standards rather than merely completing coursework [52]. Makulova et al. [51] described CBE as an educational approach centered on practical skills and occupational demands, prioritizing students’ comprehensive competency development over academic performance alone. The goal of this approach is to provide students with clear, quantifiable competency benchmarks that enable them to apply their knowledge in real-world contexts. In this study, the indicator system is designed based on this theoretical perspective, encompassing the five core domains and 28 sub-criteria. These standards assess not only students’ academic achievements but also their workplace adaptability, problem-solving skills, cross-cultural communication abilities, and practical competencies. This ensures that graduates from tourism programs can effectively navigate the industry’s dynamic and complex work environments.

(3) Constructivist learning theory (CLT): Vygotsky [53] proposed the CLT, which posits that learners construct knowledge through practice and interaction. Makulova et al. [50] extended this theory, arguing that competency-based education should further emphasize the integration of learning with real-world work scenarios. Specifically, students’ learning experiences should align with actual professional demands, enabling them to bridge the gap between acquired knowledge and workplace application, thereby enhancing their problem-solving abilities and practical skills. Therefore, this study highlights key competencies in the indicator system, including “Management Decision-Making and Analytical Skills *SC*_21_,” “Research and Investigation Skills *SC*_23_,” “Innovation and Entrepreneurship Skills *SC*_24_,” and “Crisis Management and Handling Skills *SC*_26_.” These competencies ensure that students can apply their knowledge in professional settings, thereby enhancing their employability and career competitiveness.

(4) Industry-academia collaboration and practice-oriented education: The development of undergraduate education increasingly emphasizes close cooperation between academic institutions and industries to cultivate talents that meet industry needs [54]. Makulova et al. [51] highlighted the importance of industry-academia collaboration in competency-based education. Through partnerships between universities and the tourism sector, educational institutions can gain a deeper understanding of industry demands, allowing them to adjust curricula and indicator systems accordingly. This alignment ensures that students’ competencies match contemporary professional requirements and market trends. In designing the indicator system, this study incorporates practical competencies such as “Management Decision-Making and Analytical Skills *SC*_21_,” “Research and Investigation Skills *SC*_23_,” “Innovation and Entrepreneurship Skills *SC*_24_,” and “Crisis Management and Handling Skills *SC*_26_.” These indicators emphasize the critical relationship between academia and industry, ensuring that students can seamlessly transition into the workforce upon graduation.

3. Cross-contextual comparative analysis of indicator systems.

By systematically comparing our MDM and AHP–based indicator system with established indicator systems from diverse disciplines, regions, and cultural contexts (see Table 9), we substantiate its scientific rigor and broad applicability. In engineering education [55], indicator systems emphasize structured process cycles—paralleling our *C*_1_ domain’s focus on foundational knowledge and operational workflows. Leadership competency models in U.S. hospitality [56] and Delphi-derived taxonomies in Malaysia [57] underscore interpersonal effectiveness and ethical literacy, directly reflecting our *C*_3_ and *C*_4_ criteria. IPA-based systems in Russia [58] and O*NET-informed frameworks in the United States [59] highlight sustainability, marketing, and technological adaptability—competencies embedded within our *C*_2_ and *C*_5_ domains. Finally, supervisory skill surveys in Kenya [60] confirm the universal importance of customer service and operational safety, aligning with our *SC*_13_ and *SC*_52_ indicators. By incorporating geometric aggregation of expert judgments and rigorous consistency testing, our indicator system not only integrates these cross-sectoral and cross-cultural insights but also demonstrates internal cohesion and methodological soundness. This multi-dimensional validation confirms that our indicator system is both robust and relevant across varied disciplinary, regional, and cultural settings.

**Table 9:** Comparison of the proposed evaluation system and existing systems.

Literature	Evaluation Method	Competency Dimensions	Target/Region	Differences vs. This Study/Commonalities
Crawley et al. [55]	CDIO self-assessment (5-point)	Engineering design cycle: Conceive, Design, Implement, Operate	Engineering students/Leading global technical universities	**Difference:** Focuses on engineering process skills. **Commonality:** Uses hierarchical structure and continuous improvement; parallels our *C*_1_ foundational knowledge emphasis on industry processes.
Shum et al. [56]	Questionnaire + quantitative statistics (frontline vs. director)	Leadership: Business leadership, Interpersonal leadership, Team management	Frontline and director-level hotel managers/USA	**Difference:** Emphasizes leadership layers for managerial roles. **Commonality:** Aligns with our *C*_3_ leadership & teamwork and interpersonal skills indicators (*SC*_31_, *SC*_32_).
Andrades & Dimanche [58]	Importance – Performance Analysis (IPA)	Tourism professional skills: Sustainability management, Marketing, Research	Tourism professionals/Russia	**Difference:** Prioritizes destination competitiveness via IPA. **Commonality:** Overlaps with our *C*_2_ marketing and consumer behavior (*SC*_14_) and *C*_4_ ethical/social responsibility in sustainable tourism.
Huang & Baker [59]	O*NET database SWAT analysis	Transferable skills: Work activities, Abilities, Technology skills	Entry-level hospitality/tourism workers/USA	**Difference:** Relies on large-scale secondary data. **Commonality:** Matches our *C*_2_ digital & internet skills (*SC*_25_) and crisis management (*SC*_26_) emphasis on technology and adaptability.
Francis et al. [60]	Survey + interviews + statistical testing	Supervisory, Managerial, Technical/Operational skills	Hotel supervisors/Kenya	**Difference:** Investigates education–industry disconnect in Kenya. **Commonality:** Reflects our *C*_1_ customer service (*SC*_13_) and *C*_2_ managerial skills (*SC*_16_) and first-aid/safety elements akin to our *C*_5_ first aid (*SC*_52_).
Shariff & Razak [57]	Delphi (3 rounds)	69 competencies: grouped into Workplace Environment, Personal Effectiveness, Management, Academic	Hospitality graduates/Malaysia	**Difference:** Broad four-cluster structure. **Commonality:** Both use Delphi for consensus; mirrors our *C*_3_ personal effectiveness (*SC*_33_–*SC*_36_) and *C*_4_ academic/ethical literacy (*SC*_43_) clusters.
This Study	MDM + AHP	Five criteria & 28 sub-indices (*C*_1_–*C*_5_)	Tourism students/Application-oriented universities in Fujian Province	**Unique:** Tailored for applied tourism education with local coastal and forest ecotourism focus; integrates weighting and consistency checks.

#### 5.1.2 Implications.

1. Theoretical Implications.

First, previous literature highlights the interdisciplinary nature of the tourism field and the strong linkage between university education and the tourism industry, as evidenced by Altinay and Taheri [10]. Their work shows that the growth of the global tourism industry places increasing demands on higher education institutions to train professionals who can meet industry-specific needs. Our findings build on this by emphasizing that core competencies in tourism are not merely general skills; rather, they include foundational knowledge and skills, as well as specialized knowledge and abilities directly tied to tourism operations.

Second, although some standards—such as those related to interpersonal skills, management decision analysis, or cross-cultural communication—might seem broadly applicable to humanities, social sciences, or general management, our study demonstrates their tourism-specific nature through targeted modifications. For example, in our definitions, customer service skills *SC*_13_ are explicitly tied to the unique demands of tourist engagement (such as tailored service protocols for tour guiding and hotel operations), while management skills *SC*_16_ are contextualized with reference to the operational requirements of high-star hotels and travel agencies. This tailored approach, informed by a focused literature review, curriculum analysis, and expert validation, proves that even generic indicators have been redefined to meet the unique challenges and practical realities of the tourism industry.

Third, the challenges facing tourism professional education—such as balancing the diversity of curriculum content with the need for specialized training and the inadequacy of practical teaching methods—are well documented in the literature [12,13]. These challenges reinforce the need for an evaluation system that goes beyond generic academic competencies. Our indicator system, which covers areas from tourism law and cultural creativity to crisis management tailored for tourism settings, reflects the critical need to align graduate competencies with the dynamic demands of the tourism market. This alignment is essential, as argued by Kim et al. [8], to enhance graduates’ competitiveness and employability within the industry.

Finally, our study underscores the importance of collaboration between academia and industry—an approach echoed by Vo et al. [15] and Federici et al. [27]. By developing a tourism-specific occupational skills assessment system and engaging in surveys that capture industry needs, our research ensures that the curriculum and training programs are directly aligned with the skills required in the tourism sector. This theoretical grounding confirms that even those standards that appear generic are, in fact, purposefully adapted to serve the unique needs of tourism professional education.

2. Practical implications and challenges.

(1) Practical implications.

The purpose of this study is to establish a core competency indicator system for the tourism profession and to comprehensively assess students’ performance in various professional competencies. With the help of multi-criteria decision-making methods such as MDM and AHP, the requirements and development trends of the tourism industry have been studied in depth and translated into specific core competence indicators. After extensive group discussions and expert assessment, a system of indicators for the five core competencies has been established. These core competencies comprehensively cover all aspects of the competencies required for the tourism profession and are highly recognized by the industry and the academia. Through this set of standards, the case university has revised its teaching and training program and designed corresponding courses that meet each competency, in order to better train tourism professionals who meet the needs of the industry. Following extensive group discussions and expert validation, these domains were integrated into the case university’s four-year curriculum, with particular emphasis on Fundamental Professional Knowledge and Skills (*C*_1_) and Professionally Relevant Knowledge and Skills (*C*_2_) (see Appendix C).

Tourism is a highly dynamic industry in which rapid market shifts and technological advancements continually redefine core competency requirements. Traditional four-year university curricula often lack the flexibility to respond promptly to these changes. Unlike prior research that remains largely theoretical, this study conducted in-depth field investigations and introduced a competency evaluation framework at the case university—drawing on Fujian Province’s regional characteristics—to implement a series of innovative pedagogical and practical initiatives as follows.

(A) Industry–academia collaborative specializations. In each quarter, the university convenes meetings with its partner tourism and hotel enterprises to identify emerging market trends and develop specialized innovation courses accordingly. For example, in response to recent industry hotspots, the case university and its corporate partners have launched the following programs: “AI-Driven Service Innovation Specialization”, “Tourism Blockchain Applications Specialization”, “Metaverse Innovation Specialization”, and “Green Tourism Planning Specialization”. Each specialization employs a dual-mentor model—comprising both industry executives and university faculty—to integrate the latest technological case studies and hands-on exercises into the curriculum. At the conclusion of each term, partner enterprises conduct competency-based assessments of student project deliverables to ensure seamless alignment between academic instruction and industry requirements.

(B) Rolling “Second-Classroom” credit system. Building on these specializations, the university established a rolling credit mechanism: students earn a fixed number of elective credits that are not tied to predefined course titles. Each academic year, market research and employer feedback inform the dynamic introduction of forward-looking modules. Piloted within the School of Tourism, this mechanism demonstrated positive outcomes in its first year—measured by learning achievement and employer satisfaction—and was subsequently expanded campus-wide, enabling a flexible and diversified credit-planning model.

(C) Off-campus practice bases. In collaboration with renowned regional travel and hotel chains, the university co-developed two off-campus practice bases—Tourism Planning and Hotel Operations. During winter and summer breaks, students rotate through these bases, gaining firsthand exposure to industry workflows. Practice-base mentors use the evaluation framework’s *C*_1_ and *C*_2_ indicators to quantify student performance on planning proposals and service cases, then relay the results to the Curriculum Improvement Committee for real-time course optimization. This model not only provides authentic hands-on training but also fosters a robust integration of theory and practice, significantly enhancing students’ professional competence and transition to employment within host enterprises.

(D) Tourism talent evaluation committee and quarterly forums. A campus-level Tourism Talent Evaluation Committee was established to monitor and maintain the framework’s dynamic applicability. Each quarter, a Market Trends Forum convenes graduates, frontline managers, and current students to discuss industry developments and talent needs. The Committee synthesizes these insights into quarterly reports and adjusts indicator weights and content accordingly, ensuring that the evaluation system remains closely aligned with industry evolution.

(E) Longitudinal impact assessment mechanism. To assess long-term outcomes, the university created a comprehensive graduate database recording employment organization, role, and start date. Graduates are surveyed semiannually on career progression and job competency, with specialized follow-up surveys at one, three, and five years post-graduation to evaluate the practical application of core competencies and satisfaction with the specializations and practice bases. Concurrently, partner-organization supervisors participate in annual forums to provide feedback on graduates’ skill alignment, performance, and future needs. All data are centralized in the Committee’s repository, where cross-sectional and longitudinal statistical analyses identify areas for curriculum and indicator refinement. Each year, an Annual Longitudinal Impact Assessment Report is published, and its recommendations are integrated into the subsequent cycle of specializations and practice-base programs, ensuring continuous adaptation to market and industry developments.

(2) Outcomes and recognition.

Since adopting this framework, the case university has comprehensively optimized its tourism curriculum. From 2022 onward, its Tourism Management program has maintained a graduate employment rate above 98% for three consecutive years, with over 80% of alumni still employed in tourism-related positions one year after graduation—thereby alleviating industry talent shortages and enhancing student engagement and professional identity. In 2022, the program was designated a “National First-Class Undergraduate Program” by the Ministry of Education of the People’s Republic of China. Simultaneously, it was awarded a five-star rating by China’s Higher Education Professional Evaluation Agency and, in 2023, received an A+ ranking in the Tourism Management category of the China University Subject Rankings. These accolades fully demonstrate that its teaching quality and graduate market competitiveness have reached a national leading level, establishing the program as a benchmark for peer institutions.

(3) Implementation challenges and countermeasures.

During implementation, the case university encountered several structural and operational challenges, which were addressed through targeted countermeasures as follows.

(A) Internal resistance. Faculty and administrators initially expressed concerns about increased workload, reduced autonomy, and changes to evaluation metrics. To mitigate this resistance, the university organized explanatory workshops and performance-linked incentives, and appointed “change champions” within each department to advocate for the framework.

(B) Resource constraints. Establishing specializations and practice bases required substantial administrative coordination and physical resources (e.g., facilities, transportation, equipment). The university adopted a phased “pilot-first, scale-later” approach, concentrating initial efforts within the School of Tourism and securing government and Ministry of Education grant funding to alleviate financial pressures.

(C) Faculty Skill gaps. Emphasis on digital technology, sustainable tourism, and metaverse applications presented challenges for faculty with traditional tourism backgrounds. The university implemented a dual-mentor model and an in-service training program— including industry internships and specialized workshops—to equip faculty with the necessary expertise.

(D) Longitudinal assessment execution. Designing and maintaining a robust alumni tracking system, conducting long-term surveys, and performing data analysis posed technical and organizational hurdles. In response, a dedicated office was established to manage alumni relations and employer feedback, and a partnership with the School of Information Management led to the development of a centralized data repository and analytical platform, ensuring the sustainability of the longitudinal evaluation mechanism.

By systematically addressing these challenges, the case university established a solid foundation for the sustained operation and broader dissemination of its tourism competency evaluation framework.

### 5.2 Limitations and future research recommendations

Although this study has developed a tourism professional competency indicator system with practical applicability and empirically demonstrated its feasibility, several limitations in the research process and application warrant further investigation. The specific limitations and corresponding recommendations are as follows.

1. Sample scope and geographic limitations

Due to constraints of time and resources, this study’s expert survey and pilot implementation were limited to application-oriented universities within Fujian Province. While Fujian’s representative tourism resources and multi-tiered higher education system lend a degree of generalizability, regional characteristics may nonetheless influence the universality of the findings. Future research should expand the sample to include other types of regions (e.g., inland ecological zones, coastal special economic areas) or conduct cross-national comparisons to assess the system’s transferability and adaptability across diverse cultural and geographic contexts.

2. External validity across cultures and disciplines.

The current framework focuses on tourism programs at application-oriented universities in mainland China and has not been extensively validated in other disciplines (e.g., hospitality management, cultural heritage, or even engineering education). Although this study has made preliminary comparisons with competency models and methods used in Russia, Kenya, Malaysia, and the United States—and has referred to the CDIO framework in engineering education—future work should employ empirical data to examine the system’s adaptability and generalizability across different cultural and disciplinary domains.

3. Methodological subjectivity.

This study utilized MDM and AHP for indicator development and weight assignment, effectively aggregating expert opinions but inherently relying on subjective judgments. Variations in expert composition or alternative methods could yield different indicator rankings and conclusions. Future research should consider incorporating more objective or hybrid data-processing techniques—such as fuzzy MCDM or factor analysis—or undertake multi-method comparisons to enhance the stability and reliability of results.

4. Lack of longitudinal validation.

The present research concentrated on framework construction and initial application feedback, without tracking long-term impacts on student learning outcomes or career development. It is recommended that subsequent studies establish alumni-tracking and employer-feedback mechanisms to periodically collect data on graduates’ career progression and employer satisfaction, enabling longitudinal analyses to dynamically refine the framework and strengthen its practical value.

5. Implementation challenges.

Although the practical significance section has described specific applications at the case university, the real-world rollout still faces challenges such as internal resistance to reform, resource constraints, and the need for faculty training. Future research could employ qualitative inquiries or action research to explore the critical conditions and supporting strategies for successful implementation, thereby providing transferable insights for other institutions seeking to adopt this indicator system.

### 5.3 Conclusion

The purpose of this study is to establish a “Tourism Professional Skills Evaluation System in the Application-oriented Universities” to assess the skills of application-oriented students majored in tourism from an objective point of view through a rigorous methodology and procedure. The MDM and AHP play a key role in this study, and these two methods are significant tools for establishing a “Tourism Professional Skills Evaluation System in the Application-oriented Universities”. For the sake of ensuring the scientific and objective nature of the evaluation system, this study has invited a group of experts from industry, government and academia to construct the evaluation system and to determine the basic core competences of the professional tourism discipline. Based on the findings and results of this study, several research recommendations are made as follows.

1. Continuous Optimization of the Assessment System: It is recommended that the Tourism Professional Skills Evaluation System in Application-Oriented Universities be continuously refined to keep pace with the evolving trends and needs of the tourism industry. Regular updates and revisions should be made to ensure the system remains scientifically rigorous and practically relevant.

2. Enhancement of Teaching Methods and Curriculum Design: Schools should strengthen collaboration with the tourism industry and adjust curriculum content and teaching methods in a timely manner. By integrating the key competencies identified in the five core criteria $C_{1}-C_{5}$ and their 28 subcriteria, educational programs can better cultivate both the theoretical knowledge and practical skills required in the tourism sector.

3. Strengthening the Training of Students’ Core Competencies: In addition to imparting professional knowledge and skills, schools should focus on developing students’ core competencies in areas such as interpersonal and self-development skills *C*_3_, physical fitness *C*_5_, and cultural, ethical, and legal literacy *C*_4_. Emphasizing these competencies will help students adapt and thrive in the dynamic work environment of the tourism industry.

4. Deepening Industry-Academia Collaboration Based on Competency Prioritization: Table 8 shows that subcriteria such as Fundamental Knowledge of Tourism and Hospitality (*SC*_11_, rank 1), Knowledge and Skills of Customer Service (*SC*_13_, rank 2), and Knowledge and Skills for Management Decision Analysis (*SC*_21_, rank 2) receive very high weightings. Other critical competencies with notable rankings include Management Skills (*SC*_16_, rank 4), Crisis Management and Handling (*SC*_26_, rank 5), and Tourism First Aid Skills (*SC*_52_, rank 6). Based on these findings, it is recommended that universities and industry partners develop targeted collaboration initiatives—such as specialized training workshops, joint projects, and real-world internship programs—that specifically focus on enhancing these high-priority competencies. This focused collaboration will help ensure that educational programs are directly aligned with the practical and operational needs of the tourism industry.

5. Ongoing Assessment of Graduate Employment Outcomes: Universities should implement a robust mechanism for tracking and evaluating graduate employment performance and satisfaction. By regularly collecting and analyzing employment data, institutions can continually refine teaching and training programs to ensure that graduates possess the competencies (as defined by the five criteria and 28 subcriteria) that are most valued in the tourism industry.

6. Advancing Targeted Research on Tourism Competencies: While broader academic development is valuable, our results indicate that certain subcriteria—especially those with the highest weightings (e.g., *SC*_11_, *SC*_13_,*SC*_21_, *SC*_16_, *SC*_26_, and *SC*_52_)—are critical to the success of tourism professionals. Therefore, it is recommended that future research projects concentrate on these key areas to further refine and validate the competency framework. By investigating how these competencies can be enhanced through innovative teaching methods, curriculum adjustments, and new technological applications, researchers can provide valuable insights that directly inform the continuous improvement of tourism education, ensuring it remains responsive to industry trends and demands.

## Appendix: Table A. Quartile deviation detection results of the two-round questionnaire solicitation by the modified Delphi method

**Table:** 

Criteria	Sub—Criteria	Results of Round 1			Results of Round 2		
		Mean	QD	Consistency	Mean	QD	Consistency
*C*_1_	*SC*_11_	6.438	0.435	Consistent	5.875	0.325	Consistent
	*SC*_12_	6.063	0.245	Consistent	5.375	0.345	Consistent
	*SC*_13_	6.188	0.268	Consistent	5.25	0.471	Consistent
	*SC*_14_	6.375	0.165	Consistent	5.75	0.428	Consistent
	*SC*_15_	6.063	0.245	Consistent	6.125	0.165	Consistent
	*SC*_16_	6.438	0.545	Consistent	5.25	0.787	Consistent
	*SC*_17_ ⋆	Suggested Expert Criteria			6.438	0.535	Consistent
*C*_2_	*SC*_21_	6.000	0.290	Consistent	6.125	0.29	Consistent
	*SC*_22_	5.563	0.523	Consistent	5.75	0.462	Consistent
	*SC*_23_	5.938	0.592	Consistent	5.875	0.438	Consistent
	*SC*_24_	6.313	0.362	Consistent	6.125	0.325	Consistent
	*SC*_25_	6.000	0.275	Consistent	5.875	0.242	Consistent
	*SC*_26_	6.125	0.358	Consistent	5.375	0.315	Consistent
	*SC*_27_ ⋆	Suggested Expert Criteria			5.75	0.532	Consistent
*C*_3_	*SC*_31_	6.438	0.435	Consistent	5.5	0.434	Consistent
	*SC*_32_	6.625	0.456	Consistent	5.625	0.345	Consistent
	*SC*_33_ ⋆	Experts suggest merging guidelines			5.5	0.454	Consistent
	*SC*_34_	6.813	0.127	Consistent	Consistent	0.535	Consistent
	*SC*_35_	6.750	0.135	Consistent	Consistent	0.535	Consistent
	*SC*_36_	6.563	0.216	Consistent	6.313	0.446	Consistent
	*SC*_41_ ⋆				5.563	1.685	Consistent
	*SC*_42_	6.438	0.535	Consistent	6.563	0.555	Consistent
	*SC*_43_ ⋆	Suggested Expert Criteria			6.313	0.362	Consistent
	*SC*_44_ ⋆	Suggested Expert Criteria			6.125	0.358	Consistent
*C*_4_	*SC*_51_	5.375	0.446	Consistent	5.563	0.523	Consistent
	*SC*_52_	6.375	0.235	Consistent	6.313	0.346	Consistent
	*SC*_53_	5.688	0.257	Consistent	6.625	0.155	Consistent
	*SC*_54_ ⋆	Suggested Expert Criteria			5.938	0.592	Consistent

Note: The gray part is the final criteria for obtaining expert consistency.

## Appendix: Table B. Definition of Sub-criteria for professional skills

**Table:** 

Criteria	Sub-Criteria	Define
**Fundamental professional knowledge and skills** (*C*_1_)	Fundamental Knowledge of Tourism and Hospitality (*SC*_11_)	As a student of tourism management, he/she should master the basic knowledge of the main segments of tourism and hospitality such as scenic spots, travel agencies, study tours (tourism), high-starred hotels, non-standard accommodation (family tourism, B&B, guest houses), which is the basis for working in the tourism industry.
	Skills in foreign language and conversation (*SC*_12_)	Mastering the foreign language required for work and have strong listening, speaking, reading and writing skills to serve foreign visitors without difficulties.
	Knowledge and skills of customer (job) service (*SC*_13_)	The duties, job content and qualification requirements of different jobs are different, therefore, one should master and flexibly apply the service knowledge and skills of the series of jobs engaged.
	Knowledge and Skills in Marketing and Consumer Behavior (*SC*_14_)	Marketing is the discovery of future and existing tourism markets from the perspective of meeting corporate objectives. The classical view is the 4Ps, i.e. product, price, place, and promotion. Consumer behavior refers to the various actions taken by consumers to acquire, use, and dispose of consumer goods or services, including the decision-making process that precedes and determines these actions. By focusing on the commonality and individuality of consumer behavior, a better marketing can be achieved.
	Knowledge of Tourism Administration and Regulations (*SC*_15_)	Governing by law is an integral part of the rule of law. To learn and apply the knowledge of laws and regulations involving the field of tourism such as “Tourism Law of the People’s Republic of China”, “Tourism Safety Management Measures”, “Fire Prevention Guidelines for Countryside Leisure (B&B) Buildings (for trial implementation)” and “Tourism Regulations” formulated by provincial people’s governments.
	Knowledge and skills of management (*SC*_16_)	This includes the knowledge and skills of the primary managers, mid-level managers and senior managers of tourism enterprises. Take high-starred hotel as an example, the knowledge and skills of the reception department, room services department, food and beverage department, recreation and sports departments and other departments that directly serve customers are the main criteria for the skills.
	Tourism Cultural Creativity and Planning (*SC*_17_)	In the context of aesthetic economy, the integration of tourism and cultural creative industries helps to stimulate new service products (such as “Impression Series” tourism performance) and service forms within the industry or on the periphery of the industry, creating a new tourism experience field and “unconventional environment”, which also directly promotes industrial innovation and accelerates the pace of industrial structure upgrading.
Professionally relevant knowledge and skills (*C*_2_)	Knowledge and Skills for Management Decision Analysis (*SC*_21_)	This refers to decision-making methods and logical techniques specifically applied in tourism settings, enabling managers to make informed decisions amid dynamic market conditions and industry-specific challenges.
	Knowledge and Skills of Financial Management (*SC*_22_)	This includes financial competencies tailored to tourism enterprises, such as managing procurement, inventory, revenue, operating costs, and financing specifically in the context of tourism operations.
	Knowledge and Skills for Research Surveys and Analysis (*SC*_23_)	Proficiency in research methods—such as sample surveys, in-depth interviews, and big data analysis—is essential for conducting tourism market research, assessing tourist satisfaction, and analyzing destination performance to support effective problem solving.
	Knowledge and Skills for Innovation and Entrepreneurship (*SC*_24_)	Students should develop an entrepreneurial mindset and practical innovation skills within the tourism context. This includes understanding the industry-specific opportunities, risks, and the process of launching new tourism products or services.
	Knowledge and skills of Computer and Internet (*SC*_25_)	In the digital age, tourism management students must master not only general digital tools (e.g., Excel, Word) but also specific digital competencies such as tourism big data analysis, intelligent tourism services, and digital marketing techniques tailored for the travel industry.
	Knowledge and Skills of Crisis Management and Handling (*SC*_26_)	This pertains to tourism-specific crisis management strategies, including crisis monitoring, early warning, decision-making, and response systems designed to mitigate risks unique to tourism enterprises (e.g., safety incidents at tourist sites).
	Knowledge and skills of Interdisciplinary (*SC*_27_)	This indicator emphasizes the importance of integrating knowledge from tourism business services with complementary fields such as management psychology, cross-cultural management, and digital innovation, all of which are critical for addressing the multifaceted challenges of the tourism industry.
Interpersonal and self-development skills (*C*_3_)	Leadership and teamwork skills (*SC*_31_)	Leadership requires both formal authority, using power influence (rewards and punishments), and non-power influence (character, talent, knowledge, emotions) to enhance charisma. Teamwork is the ability of a group of members with complementary strengths to help each other, work together, and work in harmony to achieve a common goal or vision. A team needs a good leader, therefore, leadership and teamwork are complementary. In tourism, effective leadership and teamwork is crucial for managing diverse teams in dynamic environments, such as coordinating tour groups or managing hotel staff, where both formal authority and personal influence are needed.
	Interpersonal skills (*SC*_32_)	The interpersonal skills are the basis for good service and management of tourism enterprises, which mainly consist of six aspects, such as interpersonal perception ability, personal memory, interpersonal understanding, interpersonal imagination, poise and expression, cooperation and coordination ability. These skills are foundational in tourism, where strong interpersonal abilities—such as perception, memory, understanding, and effective communication— directly impact service quality and customer satisfaction.
	Psychological construction and environmental adaptability (*SC*_33_)	This reflects the capacity to maintain psychological resilience and adapt to the often dynamic and seasonally fluctuating conditions of the tourism industry, ensuring effective performance under varying work environments.
	Credibility (*SC*_34_)	The degree of honesty and trustworthiness of a person is judged based on the mainstream social credibility conventions. In the tourism context, credibility—judged by honesty and trustworthiness—is vital for building customer confidence and ensuring repeat business in highly service-oriented settings.
	Social responsibility consciousness (*SC*_35_)	Social responsibility consciousness is the concrete embodiment of the perception toward the world, life, and value in the society. In addition to providing cost-effective products and services, the employees of tourism enterprises should persuade customers to “practice conservation and oppose food waste”, promote green and low-carbon travel, and do their best to help the needy and other social responsibilities. This indicator emphasizes the role of tourism professionals in promoting sustainable travel practices and community engagement, such as advocating for conservation, reducing food waste, and supporting local initiatives.
	Career planning skills (*SC*_36_)	Career planning is the process of continuous and systematic planning for a career and a life. A complete career plan consists of three elements: career orientation, goal setting and access planning. Focused on tourism careers, this involves systematic career planning, including setting clear career orientations, goals, and pathways that align with the evolving demands of the tourism industry.
Cultural, ethical and legal literacy (*C*_4_)	Literary appreciation and talent (*SC*_41_)	Literary appreciation refers to the process of understanding and emotionally experiencing works of literature and art. Talent is the skill and ability to master or reach a certain height within the scope of art after acquiring and practicing it for many years. Literature and talent can entertain both people and oneself. In tourism, an appreciation of literature and art enhances the ability to curate cultural experiences and promote local heritage, which is essential for cultural tourism and enriching visitor experiences.
	Consciousness of acting in accordance with the law (*SC*_42_)	Tourism industry and practitioners should consciously study the “Tourism Law of the People’s Republic of China”, “Labor Contract Law of the People’s Republic of China” and other relevant laws and regulations, and carry out business management and service activities involving legal issues should be handled to comply with the law.
	Knowledge and Cultivation of Professional Ethics (*SC*_43_)	The professional codes of conduct and norms that should be followed by tourism industry employees in the course of tourism-related business management and service activities, reflecting certain professional characteristics and adjusting certain professional relationships, including credibility in business, prohibition of discrimination against different religious beliefs and so on. This indicator stresses the importance of adhering to professional ethics in tourism, including fair business practices and non-discrimination, which are critical for maintaining trust in service delivery within the tourism sector.
	Knowledge of the traditional culture of the country and the nation (*SC*_44_)	Traditional culture is the “soul” and “root” of nations and countries, and is an important reference to the diversity of civilizations in the world. In the process of transnational operation or serving international tourists, tourism enterprises frequently encounter the problem of cross-cultural communication and understanding, and it will be easier to communicate across cultures if they can make use of “harmony and difference” and tolerance and mutual appreciation of Chinese traditional culture. A deep understanding of traditional culture is essential for tourism professionals, particularly when engaging with international tourists or managing cultural heritage sites, as it enables effective cross-cultural communication and enriches the tourist experience.
Physical fitness (*C*_5_)	Recreational sports or mountain sports ability (*SC*_51_)	Recreational sports mainly include different categories such as fitness, health care, recreational games and sports competitions; while mountain sports include mountaineering events, hiking traverses, overturning challenges, wilderness orienteering races, camping conferences, rock climbing races, wild cross running, mountain biking, hiking festivals, hot air ballooning, paragliding and other projects. The number of tourists who like recreational sports or mountain sports is increasing, and the employees of relevant tourism enterprises, especially coaches and service personnel, should have rich knowledge and skills of recreational or mountain sports. Tourism professionals, especially those involved in adventure or outdoor tourism, should possess specialized skills in recreational or mountain sports to ensure safety and enhance the tourist experience.
	Tourism first aid skills (*SC*_52_)	Tourism first aid skills are required when the safety of tourists’ lives and properties are endangered by a single factor or multiple factors such as tourists themselves, tourist sites or the natural environment, including but not limited to medical, earthquake, fire, water and other first aid skills.
	Endurance (*SC*_53_)	The quality of endurance refers to the body’s ability to maintain a specific intensity load or quality of action within a certain period of time. In the tourism season, the employees of tourism enterprises, especially those who directly serve tourists are required to have better endurance quality because of the longer working hours, higher work intensity and increased strain. Given the physically demanding nature of many tourism roles, particularly during peak travel seasons, employees must have high endurance to manage long hours and high-intensity workloads in dynamic tourism environments.
	Agility (*SC*_54_)	Agility is crucial for tourism staff who must respond swiftly to unexpected situations and emergencies, ensuring that service interruptions are minimized and guest safety is maintained.

## Appendix: Table C. Four-year curriculum development plan for $C_{1}$ and $C_{2}$ competencies

**Table:** 

Enhanced Competencies		Curriculum Module/Workshop
**Fundamental professional knowledge and skills** (*C*_1_)	Fundamental Knowledge of Tourism and Hospitality (*SC*_11_)	Introduction to Tourism Industry: This foundational course systematically introduces students to the tourism value chain, relevant policies and regulations, and key business operation processes. It aims to provide students with a comprehensive understanding of the industry’s overall structure and core operational mechanisms.
	Skills in foreign language and conversation (*SC*_12_)	International Tourist Reception: This course covers international hotel etiquette and foreign language communication skills. Co-taught by native-speaking instructors, it enhances students’ proficiency in listening, speaking, reading, and writing in English. During semester breaks, students participate in immersive simulation and practical training at partner hotels to reinforce their international reception skills and language adaptability through real-world applications.
	Knowledge and skills of customer (job) service (*SC*_13_)	Tourism Service Practice Workshop: Through simulated scenarios in front office operations and tour guiding, this workshop equips students with hands-on professional and workplace service skills required for various experience roles in the tourism sector.
	Knowledge and Skills in Marketing and Consumer Behavior (*SC*_14_)	Tourism Marketing: This course develops students’ ability to apply the 4Ps theory and consumer decision-making models through case studies. It fosters strategic thinking and market analysis skills critical to tourism marketing and business planning.
	Knowledge of Tourism Administration and Regulations (*SC*_15_)	Tourism Laws and Safety Management: This course provides students with a solid grounding in industry regulations, including the Tourism Law of the People’s Republic of China and relevant local policies. It prepares students to understand and apply legal compliance in tourism operations.
	Knowledge and skills of management (*SC*_16_)	Tourism Enterprise Management: This course enhances students’ skills in project planning and integrated management. The curriculum incorporates real-world operations of high-end hotels and arranges departmental rotations (e.g., front office, housekeeping, food and beverage, recreation) in partner hotels, enabling students to gain comprehensive insights into hotel operations and strengthen practical management capabilities.
	Tourism Cultural Creativity and Planning (*SC*_17_)	Cultural Creativity and Tourism Planning: This course guides students in designing thematic performances such as the “Impression Series” and developing accompanying tourism service packages. It aims to cultivate students’ creativity, integration of cultural elements, and tourism planning competencies.
**Professionally relevant knowledge and skills** (*C*_2_)	Knowledge and Skills for Management Decision Analysis (*SC*_21_)	Tourism Decision Analysis: This module integrates operations research, statistics, and multi-objective decision-making theory. Students learn to apply linear programming, multi-criteria analysis, and time-series forecasting, leveraging Python and Power BI software to simulate decision-making scenarios and perform data analytics in tourism contexts.
	Knowledge and Skills of Financial Management (*SC*_22_)	Tourism Enterprise Finance: This practicum module covers procurement, inventory management, revenue and cost control, financing strategies, and internal audit processes. It is designed to strengthen students’ practical financial management and analytical skills.
	Knowledge and Skills for Research Surveys and Analysis (*SC*_23_)	Tourism Market Research: Delivered through a project-based learning approach, this course develops students’ abilities in questionnaire design, in-depth interviewing, and big-data collection and processing. Students use SPSS and Python to conduct empirical analyses, enhancing their practical competence in market research and data interpretation.
	Knowledge and Skills for Innovation and Entrepreneurship (*SC*_24_)	Tourism Entrepreneurship Practice: In a team-based format, this course guides students to design viable business models for the tourism industry, conduct market-demand analyses, and perform risk assessments—fostering end-to-end entrepreneurial planning and execution skills.
	Knowledge and skills of Computer and Internet (*SC*_25_)	Smart Tourism Technology: This module strengthens students’ proficiency with digital tools and tourism-technology literacy. Coursework includes data processing, online marketing strategies, and hands-on big-data analytics in tourism, equipping students to apply technology effectively in smart-tourism environments.
	Knowledge and Skills of Crisis Management and Handling (*SC*_26_)	Tourism Safety and Crisis Response: Through simulation exercises, this course trains students in crisis identification, early-warning mechanisms, and on-site emergency procedures. It aims to cultivate robust risk management and emergency response capabilities.
	Knowledge and skills of Interdisciplinary (*SC*_27_)	Interdisciplinary Tourism Management: This module integrates insights from management psychology, regional cultural studies, and digital-technology applications. It develops students’ capacity for interdisciplinary analysis and application in complex tourism scenarios, preparing them to become well-rounded professionals with a holistic perspective.

## Supporting information

S1 Data (XLSX)
